# Quality of Commercially Available Manuka Honey Expressed by Pollen Composition, Diastase Activity, and Hydroxymethylfurfural Content

**DOI:** 10.3390/foods12152930

**Published:** 2023-08-02

**Authors:** Alicja Sęk, Aneta Porębska, Teresa Szczęsna

**Affiliations:** The National Institute of Horticultural Research, Konstytucji 3 Maja 1/3, 96-100 Skierniewice, Poland; aneta.porebska@inhort.pl (A.P.); teresa.szczesna@inhort.pl (T.S.)

**Keywords:** manuka honey, *Leptospermum scoparium*, melissopalynology, diastase number (DN), hydroxymethylfurfural (HMF)

## Abstract

Manuka honey plays a significant role in modern medical applications as an antibacterial, antiviral, and antibiotic agent. However, although the importance of manuka honey is well documented in the literature, information regarding its physicochemical characteristics remains limited. Moreover, so far, only a few papers address this issue in conjunction with the examination of the pollen composition of manuka honey samples. Therefore, in this study, two parameters crucial for honey quality control—the diastase number (DN) and the hydroxymethylfurfural (HMF) content—as well as the melissopalynological analysis of manuka honey, were examined. The research found a large variation in the percentage of *Leptospermum scoparium* pollen in honeys labeled and sold as manuka honeys. Furthermore, a significant proportion of these honeys was characterized by a low DN. However, since low diastase activity was not associated with low HMF content, manuka honey should not be considered as a honey with naturally low enzymatic activity. Overall, the DN and HMF content results indicate that the quality of commercially available manuka honey is questionable.

## 1. Introduction

Manuka honey is a dark monofloral honey derived from the New Zealand manuka tree (*Leptospermum scoparium*, *L. scoparium*); known for its distinctive taste and aroma, it plays a significant role in modern medical applications, especially as an antibacterial, antiviral, and antibiotic agent [1,2]. Manuka honey showed, for instance, bactericidal potency against *Pseudomonas aeruginosa* and *Staphylococcus aureus* (strains responsible for the inflammation of the mucous lining of the paranasal sinuses) and antiviral ability against Varicella zoster (the virus that causes chickenpox and shingles) [3,4]. In turn, a study of the combination of five novel antibiotics and manuka honey revealed the improved activity of these antibiotics against wound pathogens [5]. In addition, the honey from New Zealand promotes the growth of certain types of probiotics such as *Lactobacillus reuteri* and *Lactobacillus rhamnosus* [6]. In addition, manuka honey exhibits strong antioxidant and anti-inflammatory properties due to the presence of various bioactive compounds such as phenols, flavonoids, and enzymes, which makes it effective in the treatment of chronic ulcer and topical clinical inflammation [7,8,9]. Furthermore, numerous data highlight the anticancer effect of manuka honey [10,11]. A cytotoxic influence on human lung, breast, colon, and metastatic cancer cell lines, as well as on murine melanoma, colorectal carcinoma, and human hepatocarcinoma was observed [12,13,14].

Although the importance of manuka honey for medicinal purposes is well documented in the literature, information regarding its physicochemical characteristics remains limited. The European Union has established regulations governing the physicochemical parameters of honey in order to ensure its quality, authenticity, and safety [15]. These properties includes moisture content, apparent reducing sugars content, apparent sucrose content, water-insoluble solids content, ash content, acidity, diastase activity, hydroxymethylfurfural (HMF) content, and electrical conductivity [15]. Specifying the above-mentioned characteristics of honey is also intended to protect consumers against deceptive practices of sellers of this rarely cheap product. Two physicochemical parameters of honey, which deserve particular attention as they are crucial for honey quality control, are not clearly defined for manuka honey: the diastase activity (diastase number, DN) and the HMF content. Diastase (α-amylase) is one of the main enzymes found in honey; its activity level is considered an indicator of honey freshness and its proper processing [16]. Similarly, the content of HMF, a furanic compound formed when sugar-containing products are acidified or heated, informs about the quality of honey. Since a high level of HMF can be a result of adulteration with sugar additives, the HMF amount is also useful for assessing the authenticity of honey samples [17]. 

Most of the studies on the DN and HMF content in manuka honey carried out so far have not linked these experiments with melissopalynological analysis [17,18,19,20,21,22,23,24]. Only a few papers discuss this issue using these three methods [25,26,27]. However, in these studies, the results are presented for individual samples of manuka honeys without using the relevant statistics. It was found that the manuka honey with 75.8% of *L. scoparium* pollen grains contains a DN and HMF content equal to 19.03 (Schade units) and 16.42 mg·kg^−1^, respectively [25]. In turn, the honey samples with an *L. scoparium* pollen content of about 31 and 51% are characterized by a diastase activity of about 7.2 and 9.1 Schade, respectively, and by an HMF amount of 27.9 and 33.5 mg·kg^−1^, respectively [26]. Another study indicates that the manuka honey DN and HMF content was equal to 8.0 ± 1.0 Schade and 18.1 ± 3.2 mg·kg^−1^, respectively, but declared that the pollen analysis was performed; however, the percentage composition of pollen grains in the tested honey was not given [27]. Importantly, since the manuka pollen was considered over-representative and the manuka tree shares similar pollens with the kanuka bush, Moar stated that to be called manuka honey, honey must contain at least 70% of manuka pollen grains [28]. Thus, it is likely that some of the honeys mentioned should not be called manuka honey.

The problem of manuka honey falsification has been recognized earlier; in response to this issue, the New Zealand Ministry for Primary Industries reported the combination of five attributes required to distinguish manuka honey from other honey types, as well as to identify monofloral and multifloral manuka honeys [29,30]. Four of the attributes refer to the four specific compounds found in manuka honey by liquid chromatography–tandem mass spectrometry, i.e., 2′-methoxyacetophenone, 2-methoxybenzoic acid, 3-phenyllactic acid, and 4-hydroxyphenyllactic acid; the other relates to the DNA markers of manuka pollen determined by the multiplex qPCR assay [29,30]. Other studies have described the potential of compact atmospheric solids analysis probe mass spectrometry, liquid chromatography–high resolution mass spectrometry, fluorescence spectroscopy, and ^1^H NMR spectroscopy for the evaluation of manuka honey authenticity [31,32,33,34]. Another unique feature of manuka honey is its high methylglyoxal (MGO) content [35]. This compound can be formed in foods through five pathways. The first pathway is the Maillard reaction, i.e., a reaction between amino acids and reducing sugars that occurs during food storage at room temperature and during thermal processing [36]. The other pathways include the autoxidation of hexoses, the oxidation of unsaturated fatty acids in lipids, the dehydration of dihydroxyacetone (DHA), and the microbial metabolism of dihydroxyacetone phosphate [36]. In manuka honey, MGO is naturally formed as a result of the dehydration of dihydroxyacetone, a compound present in the nectar of manuka tree flowers [36]. The chemical transformation of DHA to MGO occurs in moderate heat, and the period of storage, which means that the MGO in manuka honey has already been produced in the beehive, as well as being produced in storage drums after humans collect the honey [37]. It is worth mentioning that manuka honey is frequently stored for several years without any temperature regulation [37]. In some cases, this is to intentionally increase the amount of MGO formed, especially since the MGO is thought to be responsible for the antibacterial activity of manuka honey [37,38]. It is easy to determine that advanced and often sophisticated equipment is needed to apply above described techniques used for verifying the authenticity of manuka honey; therefore, pollen analysis appears to be the most suitable first-choice method for manuka honey recognition. Thus, in this paper, using the example of the Polish market, we demonstrate the variation in the percentage of *L. scoparium* pollen in honeys labeled and sold as manuka honeys. Then, we present the DN value and HMF content based on relevant statistics. The pollen analysis results can be regarded as an alert both for consumers of manuka honey and for sellers purchasing the product from unproven suppliers, and above all for scientists who conduct research on manuka honeys without confirmation of their authenticity. In turn, the DN and HMF content results point to the questionable quality of the imported manuka honey. 

## 2. Materials and Methods

### 2.1. Chemicals

Glycerol (pure per analysis grade, ppa grade), gelatine (ppa grade), sodium hydroxide 0.1 M analytical weighed amount, sodium acetate trihydrate (ppa grade), potassium hexacyanoferrate (II) trihydrate (ppa grade), and zinc acetate dihydrate (ppa grade) were purchased from Chempur (Piekary Śląskie, Poland). The Phadebas Honey Diastase Test tablets were bought from Magle Life Sciences (Malmö, Sweden) and glacial acetic acid (ppa grade) from Pol-Aura (Zawroty, Poland). Methanol and hydroxymethylfurfural were obtained from J.T. Baker (Gliwice, Poland) and Merck (Darmstadt, Germany), respectively, and were of HPLC grade. For the analyses, distilled or ultrapure water from the Milli-Q system (Merck, Darmstadt, Germany, resistivity 18.3 MΩ cm) was used.

### 2.2. Samples

The study included thirty honey samples labeled as manuka honey available on the Polish market. All honeys had current best before dates, and the analyses were carried out immediately after obtaining the samples for testing.

### 2.3. Melissopalynological Analysis

The pollen analysis of honey was performed according to the previously described procedure with slight modifications [39]. Briefly, ten grams of honey was dissolved in 50 mL distilled water at about 50 °C. The solution was then centrifuged for 8 min (3000 rpm) and, after removing the supernatant, 50 mL of distilled water was added to the sample again and the centrifugation was repeated. After the second centrifugation, the supernatant was not completely removed, leaving a tiny amount of solution above the sediment. The residue was mixed and placed on a microscope slide. Next, the slide was dried and secured with glycerol/gelatine and a coverslip. The collected material was analyzed using an Olympus BX41 microscope (Olympus America, PA, USA)) at 400× magnification. At least 300 consecutive pollen grains of nectar-producing plants were determined on each microscopic slide, and then the percentage content of *L. scoparium* pollen was calculated. Two repetitions were made for each honey sample and the final result is presented as an average of two results not differing between individual determinations by more than 5%, and rounded to the whole unit (%).

### 2.4. Diastase Analysis

The diastatic activity was determined by the Phadebas method in which the α-amylase activity is expressed as the diastase number, and is reported in Schade units [40]. One Schade unit corresponds to the enzyme activity contained in 1 g of honey, which can hydrolyze 0.01 g of starch in 1 hour at 40 °C. The procedure was as follows: first, 1 g of the analyzed honey was weighed, transferred to a 100 mL volumetric flask, and filled up to its volume with 0.1 M acetate buffer at pH = 5.2. Then, 5 milliliters of the sample was transferred to the test tube, placed in a water bath at 40 °C, and after 15 min a Phadebas tablet was added to the solution. The solution was mixed and heated again in a water bath for 30 min. After this time, 1 mL of 0.5 M sodium hydroxide solution was added to complete the enzymatic reaction. Next, the solution was filtered through a filter paper (φ = 70 mm) and the absorbance at 620 nm was measured using a Specord 200 spectrophotometer (Analytic Jena, Jena, Germany) as it is in proportion to the enzyme activity in the analyzed honey sample. The measurements were collected in triplicate and the final result is their average.

### 2.5. Hydroxymethylfurfural Analysis

The HMF content in the analyzed honey samples was determined chromatographically with the application of a Knauer HPLC system (Knauer GmbH, Berlin, Germany) equipped with an UV K-2501 detector and with a reversed-phase C-18 Vertex Plus Eurospher column (BGB Analytik Vertrieb GmbH, Lörrach, Germany), as described previously [41]. The internal diameter of column was 4 mm, length 250 mm, and particle size 5 μm. The column and detector were placed on a thermostat at 30 °C. As a mobile phase, a mixture of methanol and deionized water (10:90, *v*/*v*) was used, and the flow rate was 1 mL·min^−1^. For the quantitative analysis of HMF, the external standard method was applied. The determinations were carried out in triplicate. Honey samples were prepared based on the European Honey Commission procedure [40].

## 3. Results and Discussion

**Figure 1:**
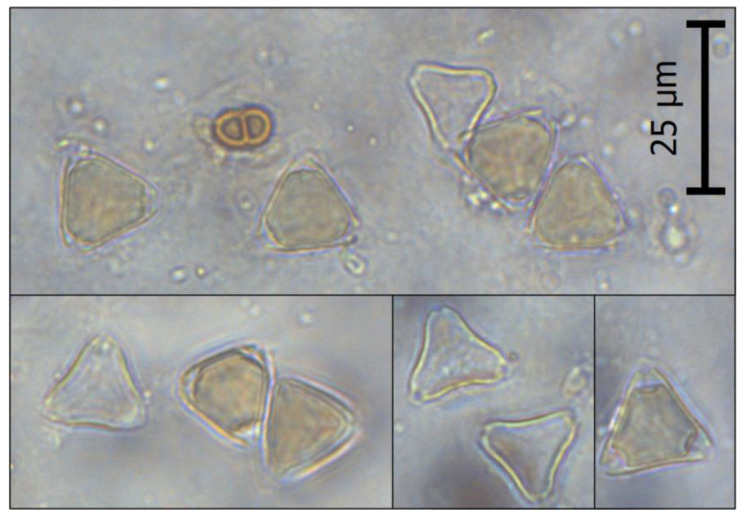
Microscopic image of *L. scoparium* pollen under 400× magnification.

The first stage of the research included the melissopalynological analysis of the examined honey samples. For each honey, at least 300 consecutive pollen grains of nectar-producing plants, including *L. scoparium* pollen, were identified and counted, and then the percentage of *L. scoparium* pollen content was calculated on this basis. The microscopic image of *L. scoparium* pollen found in the analyzed honeys is presented in Figure 1. As can be seen, the pollen grain is small (~15–20 µm), triangular in polar view, isopolar, and tricolporate, which is consistent with the literature data [42,43]. It is also worth noting that the sides of the pollen sometimes appear concave, while the angles appear extended [43]. Most of the studies to date report a significant resemblance between manuka and kanuka pollen; consequently, melissopalynological studies tend to combine them [28,43,44]. Despite its imperfections, this method is still useful as it allows for the simple verification of the presence and quantity of manuka-like pollen in a honey sample.

The results obtained from the pollen analysis of thirty honey samples labeled as manuka honeys are listed in Table 1. As shown, the percentage of *L. scoparium* pollen grains in the analyzed honeys varied from 45 to 90%. Importantly, as much as 47% of the tested honeys indicate a low content of manuka tree pollen, lower than the Moar’s limit (70%) for honey to be called manuka honey [28]. The obtained results point to the wide diversity in the manuka pollen composition of commercially available manuka honeys, and simultaneously prove the importance of the melissopalynological analysis of purchased honeys, especially in order to verify the authenticity of honey without the use of advanced methods. 

**Figure 2:**
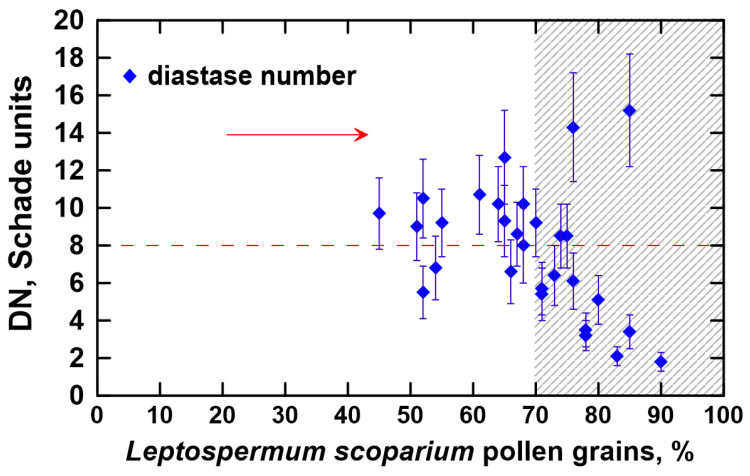
The diastase number (DN) in the analyzed honeys.

In the next step, the activity level of α-amylase was determined and plotted in Figure 2. While the DN was estimated for all analyzed honey samples, we focus our discussions on the results obtained for the melissopalynologically classified manuka honey samples; that is, honeys that contain at least 70% of manuka pollen (shaded area of the graph). As illustrated, the diastase level ranged from 1.8 to 15.2 Schade units, but was mostly lower than 8 Schade units. According to the European Directive that governs the standards for honey sold in the European market, a minimum diastase level of 8 Schade units is necessary for honey to be deemed of good quality and acceptable; thus, the vast majority of the analyzed manuka honeys do not meet this requirement [15]. In fact, merely five manuka honeys (31%) had a diastase activity higher than 8 Schade units. Nevertheless, honeys with naturally low enzymatic activity are known, such as *Acacia*, *Becium grandiflorum*, *Croton macrostachyus*, *Eucalyptus globulus*, *Hypoestes*, *Leucas abyssinica*, *Schefflera abyssinica*, *Syzygium guineense*, and *Vernonia amygdalina* monofloral honeys [15,45]. Such honeys are characterized by a DN value between 3 and 8 Schade units and an HMF content lower than 15 mg·kg^−1^ [15,46]. 

**Figure 3:**
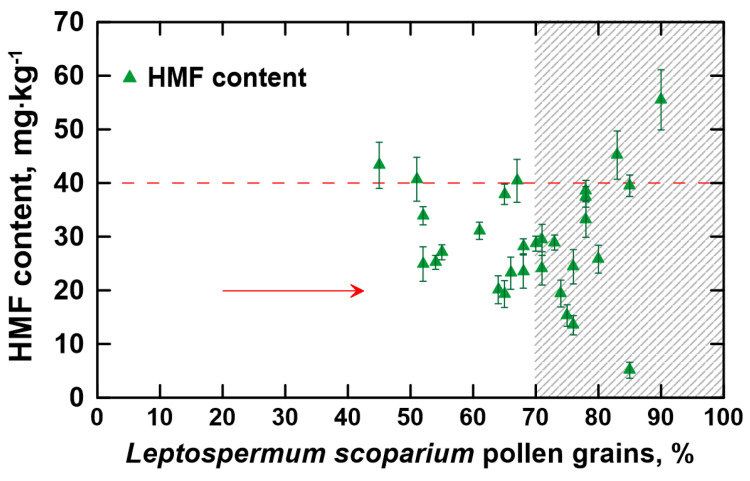
The hydroxymethylfurfural (HMF) content in the analyzed honeys.

Figure 3 provides the HMF content in the manuka honey samples evaluated in this study. Only two melissopalynologically classified manuka honeys showed the HMF content below 15 mg·kg^−1^, more precisely 13.5 ± 1.8 mg·kg^−1^ (when the percentage of *L. scoparium* pollen grains was 76%) and 5.1 ± 1.5 mg·kg^−1^ (when the percentage of *L. scoparium* pollen grains was 85%). However, the DN for these samples was 14.3 ± 2.9 Schade units and 15.2 ± 3.0 Schade units, respectively. This denotes that the low level of HMF present in this honey does not coincide with a low level of diastase activity, which would typically be expected in honeys with naturally low enzymatic activity. Thus, even though manuka honey is most often characterized by a low level of α-amylase activity, it should not be considered a honey with intrinsically low enzyme activity as it does not fulfill the second condition for such honeys [15]. The low DN may result from thermal treatments or long-term storage, as well as from storage under inappropriate conditions [47].

Nearly all of the manuka honey samples with a high content of manuka pollen (shaded area in Figure 3) satisfy the maximum limit of HMF content allowed by the European Directive, which is 40 mg·kg^−1^ [15]. Specifically, the HMF content ranged from 5.1 to 55.5 mg·kg^−1^ and two samples (13%) exceeded the permissible limit. These two samples had HMF values of 45.2 ± 4.5 mg·kg^−1^ and 55.5 ± 5.6 mg·kg^−1^, and were characterized by a DN equal to 2.1 ± 0.5 Schade units and 1.8 ± 0.5 Schade units, respectively. Such a combination of HMF content and DN clearly indicates that the honeys were of poor quality, presumably due to overheating or improper/prolonged storage [48]. According to European regulations, these honeys should be withdrawn from sale, especially since the HMF impact on human health is ambiguous [15,41,49,50]. Other melissopalynologically classified manuka honeys, based on the HMF content, may have been released for consumption. 

**Figure 4:**
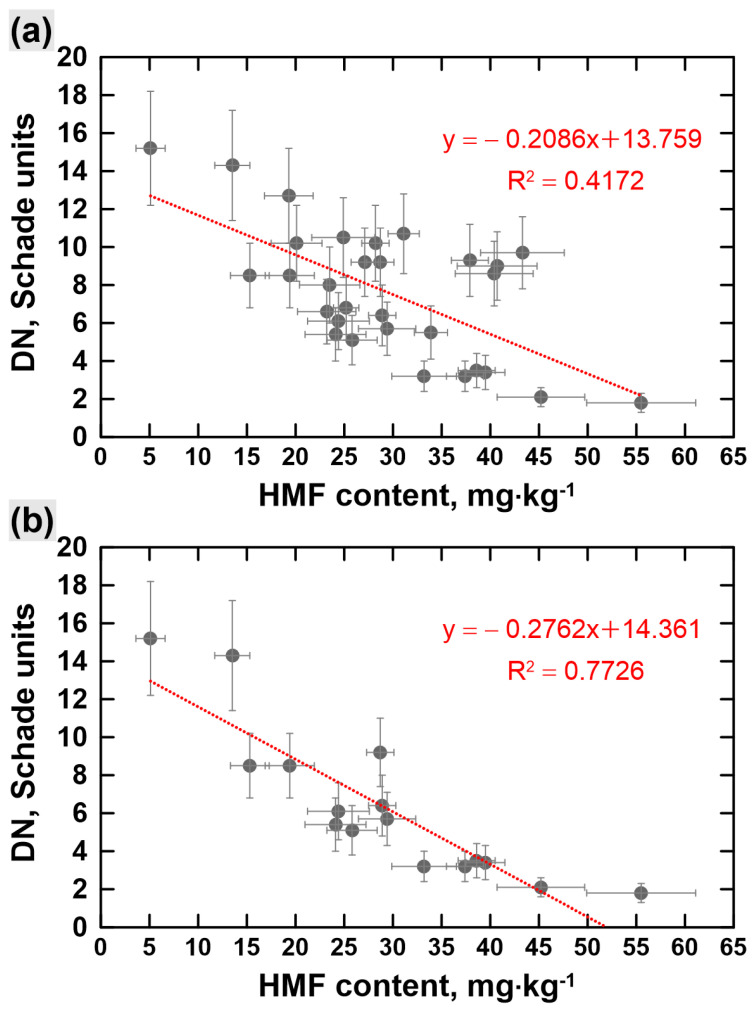
The correlation between the diastase number (DN) and hydroxymethylfurfural (HMF) content obtained for: (**a**) thirty honey samples labeled and sold as manuka honeys; (**b**) the part of the examined manuka honeys that contains at least 70% of manuka pollen.

To gain further insight into the characteristic of commercially available manuka honeys, the correlation between the DN value and HMF content was analyzed. As can be seen in Figure 4, showing a comparison of the regression graph between the diastatic activity and HMF content obtained for thirty honey samples labeled and sold as manuka honeys (Figure 4a) and for the manuka honeys with a high content of manuka pollen, that is, honeys that contain at least 70% of manuka pollen (Figure 4b); in both cases, the DN presents a substantial negative relationship with HMF. Regression equations for diastase activity to HMF content for all analyzed honey samples and for melissopalynologically classified manuka honeys were y = −0.2086x + 13.759 and y =−0.2762x + 14.361, respectively. This shows that the negative regression is even more significant when the manuka honeys with a high content of manuka pollen are considered. Notably, the obtained dependencies are in good agreement with the knowledge that a high HMF content is a marker of excessive heating or improper storage of honey, which in turn causes a decrease in the enzymatic activity. Similar behavior was reported in the study of the Ethiopian monofloral honeys [45]. As a result of the regression analysis of these honeys, the following regression equation for DN to HMF was obtained: y = −0.1389x + 6.3701 [45]. In addition, the study of honeys from Bosnia and Herzegovina and Algeria also revealed a negative correlation between the diastase activity and HMF content [51,52]. 

A concise summary of the results obtained for the melissopalynologically classified manuka honeys is presented in Table 2. As depicted, the average percentage of *L. scoparium* pollen grains in the analyzed honeys was 77.7 ± 5.7%, while the mean DN and the mean HMF content were 6.4 ± 4.0 Schade units and 29.0 ± 12.7 mg·kg^−1^, respectively. These results indicate there is a wide variety of manuka honeys available on the Polish market in terms of enzymatic activity and HMF content. In addition, a significant part of these honeys do not meet the requirements of the European Directive authorizing honey for use, mainly due to the low DN. The obtained results suggest that the analyzed manuka honeys were stored under inappropriate conditions for long periods of time or were intentionally heated, for example in order to achieve a higher content of methylglyoxal, a compound believed to be the predominant antibacterial constituent of manuka honey [53]. Thus, the physicochemical quality of these imported honeys is questionable. 

## 4. Conclusions

In the paper, thirty honey samples labeled and sold as manuka honey were tested for their pollen composition, diastase number, and HMF content. The obtained results indicate the large diversity of manuka honey available on the Polish market both in terms of the percentage of *L. scoparium* pollen grains and the enzymatic activity or HMF content. The most striking observation from the experiments is that, according to Moar’s statement and based on the pollen analysis, almost half of the analyzed honeys should not be classified as manuka honeys since their *L. scoparium* pollen content did not meet the required minimum. Presumably, the Polish market is not the only market facing such a problem. Thus, the presented results can be regarded as a general warning for consumers of manuka honey and for sellers purchasing the product from unverified suppliers, and above all for scientists conducting research on manuka honey without confirmation of its authenticity. It is also worth emphasizing that a significant proportion of the analyzed honeys had a low DN, lower than the European Directive limit for the honey to be approved for use. The low diastase activity was not associated with the low HMF content, and therefore it would be incorrect to assume that manuka honey naturally has low enzymatic activity. The low value of DN and relatively high HMF content indicate, in turn, the flawed physicochemical quality of these commercially available honeys. The present research encourages further study of natural (without any additional processing) and commercially available manuka honeys. It is reasonable, for example, to extend the research by using other hallmarks of manuka honeys in order to establish their authenticity or adulteration on the basis of further scientific evidence.

## Figures and Tables

**Table 1:** The percentage of *L. scoparium* (*LS*) pollen grains in the analyzed honeys.

Sample Number	*LS* Pollen Grains
1.	45%
2.	51%
3.	52%
4.	52%
5.	54%
6.	55%
7.	61%
8.	64%
9.	65%
10.	65%
11.	66%
12.	67%
13.	68%
14.	68%
15.	70%
16.	71%
17.	71%
18.	73%
19.	74%
20.	75%
21.	76%
22.	76%
23.	78%
24.	78%
25.	78%
26.	80%
27.	83%
28.	85%
29.	85%
30.	90%

**Table 2:** Summary of the characteristics of the melissopalynologically classified manuka honeys.

Parameter	Unit	Min	Max	Mean	SD
*L. scoparium* pollen	%	70.0	90.0	77.7	5.7
Diastase number	Schade	1.8	15.2	6.4	4.0
HMF content	mg·kg^−1^	5.1	55.5	29.0	12.7

SD—Standard Deviation.

## Data Availability

All data generated or analyzed during this study are available from the corresponding author on reasonable request.
